# Impaired Frontoparietal Connectivity in Traumatic Individuals with Disorders of Consciousness: A Dynamic Brain Network Analysis

**DOI:** 10.14336/AD.2019.0606

**Published:** 2020-03-09

**Authors:** Min Wu, Fali Li, Yuehao Wu, Tieying Zhang, Jian Gao, Peng Xu, Benyan Luo

**Affiliations:** ^1^Department of Neurology & Brain Medical Centre, First Affiliated Hospital, School of Medicine, Zhejiang University, Hangzhou, China.; ^2^The Clinical Hospital of Chengdu Brain Science Institute, Key Lab for NeuroInformation, University of Electronic Science and Technology of China, Chengdu, China.; ^3^Department of Rehabilitation, Hangzhou Hospital of Zhejiang Armed Police Corps, Hangzhou, China.

**Keywords:** event-related potentials, consciousness, traumatic brain injury, frontoparietal network

## Abstract

Recent advances in neuroimaging have demonstrated that patients with disorders of consciousness (DOC) may retain residual consciousness through activation of a complex functional brain network. However, an understanding of the hierarchy of residual consciousness and dynamic network connectivity in DOC patients is lacking. This study aimed to investigate residual consciousness and the dynamics of neural processing in DOC patients. We included 42 patients with DOC, categorized by aetiology. Event-related potentials combined with time-varying electroencephalography networks were used to probe affective consciousness in DOC and examine the related network mechanisms. The results showed an obvious frontal P3a component among patients in minimally conscious state (MCS), while a prominent N1 was observed in unresponsive wakefulness syndrome (UWS). No late positive potential (LPP) was detected in these patients. Next, we divided the results by aetiology. Patients with nontraumatic injury presented an obvious frontal P3a response compared to those with traumatic injury. With respect to the dynamic network mechanism, patients with UWS, both with and without trauma, exhibited impaired frontoparietal network connectivity during the middle to late emotion processing period (P3a and LPP). Surprisingly, unconscious post-traumatic patients had an evident deficit in top-down connectivity. This, it appears that early automatic sensory identification is preserved in UWS and that exogenous attention was preserved even in MCS. However, high-level cognitive abilities were severely attenuated in unconscious patients. We also speculate that reduced frontoparietal connectivity may be useful as a biomarker to distinguish patients in an MCS from those with UWS given the same aetiology.

Disorders of consciousness (DOC), which include a spectrum of pathological states of consciousness, can be subdivided into coma, unresponsive wakefulness syndrome (UWS), and the minimally conscious state (MCS). UWS is characterized by preserved autonomic functions but no self-awareness or environmental awareness [1]. In MCS, clear yet partial and fluctuating signs of awareness are partially preserved [2]. At present, characterizing these covert abilities in individuals with DOC is crucial for their diagnosis, clinical management, and prognosis.

The last decade has seen significant advances in the application of neuroimaging technologies and electrophysiology-based assessments to accurately diagnose DOC in the absence of outward responsiveness [3, 4]. However, due to the use of various stimulation paradigms, the results obtained by neuroimaging are heterogeneous. Notably, stimuli with emotional content (e.g., the patient’s own name, music, etc.) might be more effective than the traditional sine tones in unmasking covert awareness [5], since the processing of emotionally significant stimuli is prioritized [6]. Therefore, the use of emotional stimuli may help improve the detection of residual consciousness in DOC patients. Physiologically, the brain processes emotional sound in a very efficient way, which usually takes tens of milliseconds [7]. fMRI (functional magnetic resonance imaging) shows excellent spatial resolution and is widely used in neuroscience; however, this technique relies upon the haemodynamic response and has insufficient temporal resolution to capture rapid emotion processing. Electro-encephalography (EEG), given its millisecond-level resolution, is a suitable option for investigating the brain response associated with emotion regulation. ERPs (event-related potentials), which are electrophysiological correlates of neural impulses time-locked to a stimulus, can reflect the synchronous neural activity that underlies information processing. Indeed, ERP evidence demonstrated that DOC patients successfully maintained varying degrees of residual awareness [8].

Current evidence suggests that a global hallmark of impaired consciousness caused by brain damage is profound disruption of functional connectivity. For example, investigations have reported that disrupted default mode network (DMN) activity is related to loss of consciousness, and Demertzi further reported on auditory network differences between MCS and UWS patients [9-11]. Nonetheless, recent research has shown that brain functional connectivity is not static but rather exhibits spontaneous fluctuations over time [12, 13]. However, these fluctuating network connections, especially the dynamic time-varying connections in various stages of emotional sound processing, are unclear, and understanding this dynamic network mechanism may help elucidate the impairment of information processing in DOC [14]. The adaptive directed transfer function (ADTF, also termed the time-varying DTF) has been shown to be useful for capturing dynamic network patterns that correspond to distinct stages of information processing [15, 16]. In the present study, we applied the ADTF methodology to assess DOC patients’ underlying pathological states of consciousness with the aim of obtaining spatiotemporal network information. This information may be valuable for improved understanding of the different dysfunctional connectivity patterns in UWS and MCS patients. Moreover, information on the various relationships between neural injury patterns and functional disconnections is limited. Therefore, we examined a large sample of patients with traumatic and nontraumatic DOC to ascertain the relevance of aetiology and network connectivity and to determine the mechanism of individuals’ brain injuries.

## MATERIALS AND METHODS

### Patients

A total of 42 patients (20 UWS, 22 MCS) in the Department of Rehabilitation at Hangzhou Hospital of Zhejiang CAPR were included in our study. All subjects met the study inclusion criteria: (1) no centrally acting drugs; (2) no neuromuscular function blockers and no sedation within 24 hours prior to the study; (3) periods of spontaneous eye opening; and (4) diagnosis of UWS or MCS based on the Coma Recovery Scale-Revised (CRS-R) [17]. Structural brain images were obtained beforehand using a magnetic resonance imaging (MRI) scanner for all patients, except two patients with metallic cerebral implants or pacemakers, who were assessed with computed tomography (CT). Patients with significant brain tissue defects, more than 30% of total brain volume were excluded [18]. Twenty-four healthy, age-matched, right-handed volunteers also participated in the experiment. One healthy control and 10 DOC patients were excluded due to poor EEG data. Of the remaining 32 patients, 18 met the diagnostic criteria defining UWS (10 traumatic; 12 male; age range 26 to 75 years, mean 52 years), and 14 patients met the diagnostic criteria defining an MCS (6 traumatic; 9 male; age range 31 to 73 years, mean 56 years). All demographic and clinical characteristics of the enrolled patients are shown in Table 1.

### Standard protocol approval, registration, and patient consent

The trial was conducted in accordance with the Declaration of Helsinki. Written informed consent was obtained from the legal representative of each patient. This study was approved by the Ethical Committee of the First Affiliated Hospital, School of Medicine, Zhejiang University, and by Hangzhou Hospital of Zhejiang CAPR. Moreover, the clinical study has been registered at https://clinicaltrials.gov under the registration number NCT03385291.

**Table 1:** The characteristic data of patients in DOC.

Patient	clinical diagnosis	gender/age	etiology	Lesions (CT or MRI)	Month since injury	CRS-R sub-scores	CRS-R total scores
01	MCS	M/40	Trauma	Bilateral frontal, temporal and right parietal, occipital lobe lesions	7.1	1 0 3 0 0 2	6
02	MCS	M/67	Hemorrhage	Bilateral frontal, temporal lobe lesions	3.0	0 0 3 0 0 2	5
03	MCS	F/72	Hemorrhage	Right frontal, temporal lobe, basal ganglia and brain stem lesions	6.1	1 1 3 0 0 2	7
04	MCS	M/70	Trauma	Bilateral frontal, temporal lobe lesions	2.2	0 1 4 0 0 2	7
05	MCS	F/44	Anoxia	Diffuse demyelination	3.1	2 3 1 1 0 2	9
06	MCS	M/31	Hemorrhage	Brain stem lesions	10.8	1 2 3 0 0 2	8
07	MCS	M/73	Hemorrhage	Brain stem and cerebellum lesions	4.0	2 3 4 0 0 3	12
08	MCS	M/43	Trauma	Left temporal, parietal lobe lesions	3.2	1 2 3 0 0 2	8
09	MCS	F/67	Trauma	Bilateral frontal lobe and left parietal lobe lesions	2.5	1 0 2 1 0 2	6
10	MCS	F/68	Hemorrhage	Left basal ganglia lesions	2.2	2 2 3 0 0 3	10
11	MCS	M/60	Hemorrhage	SAH	2.2	4 4 5 2 1 3	19
12	MCS	M/61	Hemorrhage	Right basal ganglia lesions	2.5	2 3 2 0 0 2	9
13	MCS	M/45	Trauma	Bilateral frontal lobe and left parietal lobe lesions	2.7	2 3 2 0 0 2	9
14	MCS	F/48	Trauma	Bilateral frontal lobe and left parietal lobe lesions	6.3	2 2 3 0 0 2	9
15	VS	M/44	Anoxia	Diffuse demyelination	7.3	1 0 1 0 0 2	4
16	VS	M/42	Anoxia	Diffuse demyelination	7.1	0 0 1 1 0 2	4
17	VS	M/65	Trauma	Left frontal and parietal lobe lesions	3.0	0 1 0 0 0 2	3
18	VS	M/34	Hemorrhage	Right temporal, parietal and occipital lobe and left frontal lobe lesions	0.7	0 0 1 0 0 2	3
19	VS	F/26	Trauma	Bilateral frontal and temporal lesions	2.1	2 1 2 0 0 2	7
20	VS	M/33	Trauma	Diffuse demyelination	6.1	0 0 1 0 0 2	3
21	VS	M/59	Trauma	Left frontal and temporal lobe, basal ganglia lesions	2.2	0 1 1 0 0 2	4
22	VS	M/62	Trauma	Right frontal and temporal lobe lesions	4.4	0 0 2 0 0 2	4
23	VS	M/52	Trauma	SAH, right temporal lobe lesions	2.5	1 1 2 0 0 2	6
24	VS	F/55	Hemorrhage	left frontal and temporal lesion	1.6	1 1 2 0 0 2	6
25	VS	M/35	Hemorrhage	Brain stem lesion	6.0	0 0 2 0 0 2	4
26	VS	F/49	Hemorrhage	Brain stem lesion	3.2	0 0 2 0 0 2	4
27	VS	M/54	Trauma	SAH	1.7	2 1 2 0 0 2	7
28	VS	F/67	Trauma	Right temporal lobe lesions	5.9	0 1 1 0 0 2	4
29	VS	F/53	Hemorrhage	SAH	1.4	2 1 2 0 0 3	8
30	VS	M/69	Hemorrhage	Brain stem lesion	2.0	0 1 2 1 0 2	6
31	VS	F/63	Trauma	Right temporal lobe lesions	4.0	0 1 2 0 0 2	5
32	VS	M/75	Trauma	Left frontal and temporal lobe lesions	2.4	2 1 2 2 0 2	9

CRS-R = Coma Recovery Scale-Revised; Six subscales score of CRS-R indicating the assessment of auditory, visual, motor, verbal, communication functions and arousal. SAH: subarachnoid hemorrhage

### ERP paradigm

The vocal stimuli were binaurally delivered to the participants at a maximum intensity of 90 dB using a passive auditory oddball paradigm. The standard stimulus was a meaningless neutral sound (namely, the interjection “ah”), while the deviant stimulus was the same sound with positive or negative affective prosody. These stimuli, uttered by four different voices, were chosen from the validated battery of vocal emotional expressions [19]. Each stimulus lasted 700 ms and was followed by an inter-stimulus interval of 1500 ms. Each of the 4 blocks contained a total of 110 stimuli in the same voice, with 86 standards, 12 happy deviants and 12 sad deviants in each of the 4 blocks. These deviant sounds were presented in a randomly permuted order, ensuring that the same sound was not presented multiple times in quick succession.

### EEG recording

The EEG was recorded using a 64-channel BrainCap (Brain Products GmbH, Munich, Germany) in the standard 10-20 system. All EEG electrodes were referenced online to FCz. A vertical electro-oculogram (EOG) was recorded supra-orbitally from the left eye, and a horizontal EOG was recorded from the right orbital rim. The impedance of all electrodes was kept below 10 k?, and 50 Hz was notched. The EEG and EOG signals were amplified using a DC 1000 Hz bandpass filter and were continuously sampled at 500 Hz/channel.

**Figure 1.**
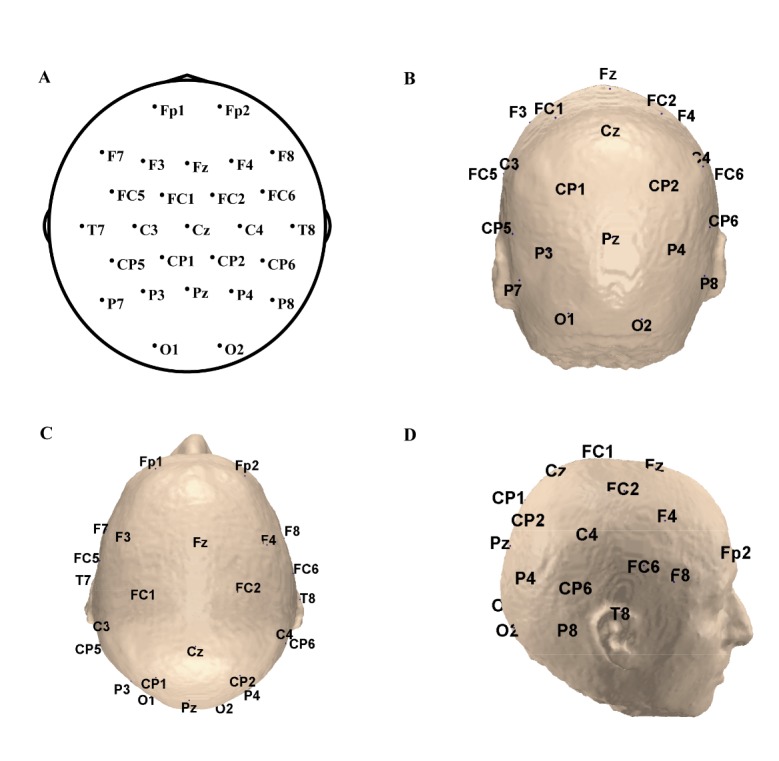
The spatial locations of the selected electrodes. (A) The electrodes used for dynamic network analysis. (B-D) The spatial locations of the electrodes: (B) back, (C) upper, and (D) right.

### EEG data processing

The continuous EEG was re-referenced to the average of the bilateral mastoid electrodes TP9 and TP10. A basic finite impulse response (FIR) filter between 0.1 and 30 Hz was used to block the DC offset and reject high-frequency noise. Independent component analysis (ICA) was performed to remove electro-oculogram artefacts, and the EEG signals were then reconstructed from the remaining components. We extracted trials spanning from -200 to 1000 ms, with the stimulus onset defined as zero, and we baseline corrected all trials to their mean voltage from -200 to 0 ms. We also excluded trials with amplitudes more than 3 standard deviations from the mean.

To lower the effect of volume conduction, we selected a sparse array of 27 electrodes and used them in a time-varying network analysis (Fig. 1) [20, 21]. For each subject, all of the remaining artefact-free trials during emotional processing were further used to construct time-varying networks with the trial-by-trial ADTF. The ADTF, as an extension of the directed transfer function (DTF), is used to extract the directional information flow between brain structures and is considered as a type of multivariate Granger causality. The ADTF is derived from the time-varying coefficients obtained from a multivariate adaptive autoregressive (MVAAR) model with the Kalman filter algorithm. In the current study, to quantitatively evaluate the directed information flow between each pair of electrodes, we applied the ADTF to all of the remaining artefact-free trials for each patient, and we averaged the constructed ADTF networks trial by trial across all trials, resulting in a final time-varying network for each patient [21, 22]. Trial-by-trial ADTF averaging can remove unstable patterns caused by noise while conserving the intrinsic time variation [15]. The details of the time-varying multivariate adaptive autoregressive (TV-MVAAR) model and ADTF method are provided in the supplementary methods.

### Network properties

The clustering coefficient (*C*), global efficiency (*Ge*), local efficiency (*Le*), and characteristic path length (*L*) are four commonly used network properties. *C* and *Le* indicate the local connectedness of a graph; *L* and *Ge* index the global connectedness. Higher values of *C, Ge*, and *Le* indicate stronger connectivity, while *L* has the opposite relationship with connectivity strength. The specific definitions of these properties are as follows:

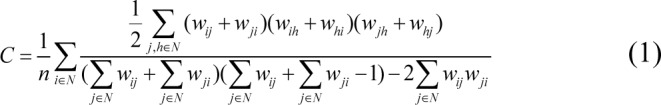


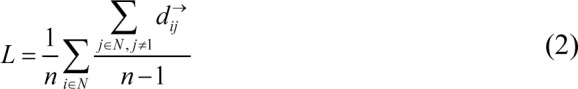


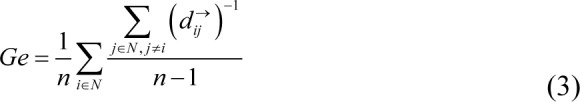


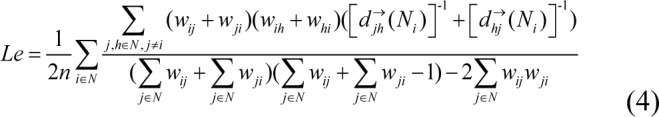


Here, *w_ij_* and 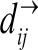


### Statistical analyses

Mass univariate analysis with a t-test at each time point for each condition was used within the EEGLAB study framework to capture the precise moments in which different neural responses emerge [23]. In our current research, a two-tailed t-test was performed at each time point from 0 ms to 1000 ms. This type of mass univariate analysis, consisting of thousands of statistical tests, is one of the most useful analyses for tracking subtle differences in rapidly changing EEG signals. In contrast to the commonly used ANOVA, which requires investigators to know beforehand approximately when an effect will occur, this approach can not only detect the expected effects with sufficient temporal resolution but also reveal several unexpected ERP components; therefore, mass univariate analysis ?is a useful method for a novel protocol in which the timing of effects is unknown beforehand [23, 24].

For time-variant network analysis, two-tailed t-tests were performed. The edges with significantly strong connections (*P* < 0.05) between two groups were retained to construct the time-varying network. Furthermore, to assess the relationships between patients’ behavioural presentations and cortical activity patterns, we calculated Pearson’s correlation coefficients between CRS-R scores and network properties.

## RESULTS

### ERP results

**Figure 2.**
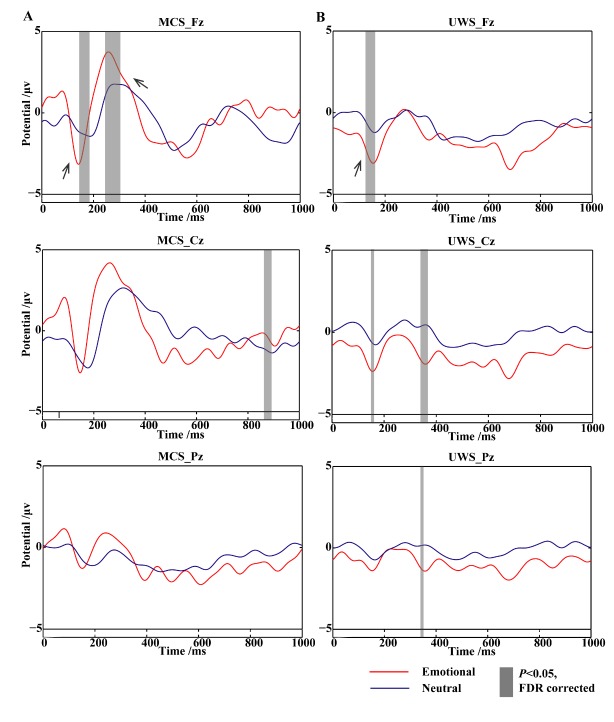
Grand average ERP to target stimuli at electrodes Fz, Cz, and Pz. (A) An obvious N1 and P3a evoked by emotional sound in MCS patients. (B) A significant emotion-evoked N1 in UWS subjects (*P*<0.05, FDR corrected).

Figure 2 and Supplementary Figure 1 show the ERP waveforms at different latencies in the emotional paradigm: the N1 waveforms (a negative dip between 100 and 200 ms at electrode Fz), the P3a waveforms (a positive deflection peaking approximately 300 ms after the stimulus at electrode Fz), and the late positive potential (LPP, a long positive elevation between 400 and 1000 ms in the centroparietal area, as shown in Figure S1). Many previous ERP studies specifically assessing emotional processing have focused on the LPP, a midline ERP that lasts for several hundred milliseconds [25]. The grey bars show the regions with significant differences between conditions (*P* < 0.05, FDR corrected). The results from healthy controls are presented in the supplementary materials (Supplementary Fig. 1).

As for DOC patients, the N1 component (and no LPP) was observed in both UWS and MCS patients. An emotion-evoked P3a was present in the MCS group (Figure 2A). However, in the UWS groups, none of the middle to late ERP components (P3a and LPP) significantly differed between the neutral and emotional settings, whereas the amplitude of N1 was increased during emotion processing (Fig. 2B).

Since the patients showed a detectable brain response to emotional stimulation, as indicated by the P3a in MCS and N1 in UWS patients, the following sections focus on exploring the neural mechanisms using emotion-inducing protocols, which involve more complex networks and plasticity mechanisms.

**Figure 3.**
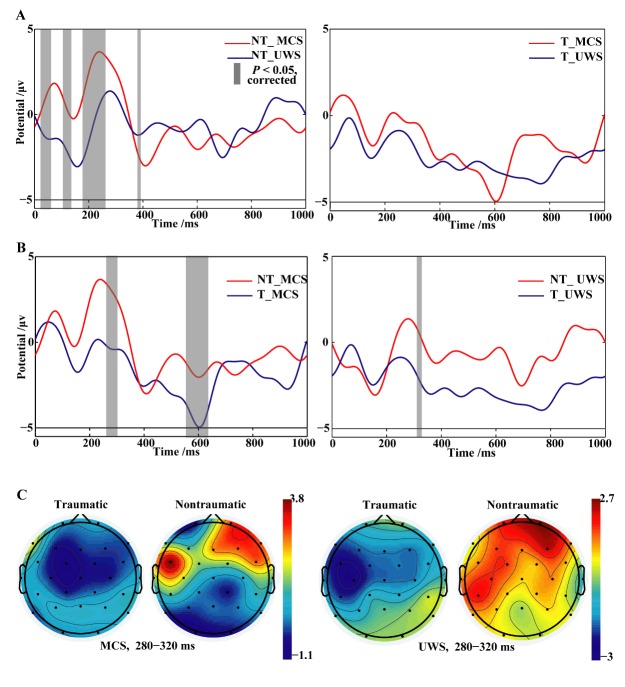
Grand average ERP waveforms at electrode Fz. (A) Both traumatic and nontraumatic MCS patients had increased P3a amplitudes at the frontal-central electrode. (B) The waveform difference between traumatic and nontraumatic groups with the same diagnosis. P3a amplitudes were enhanced in the nontraumatic participants compared with the traumatic participants. The grey bars indicate regions of significant difference between conditions (*P* <0.05, FDR corrected). (C) The scalp topography of P3a. Positive activation was detected between 280 ms and 320 ms in frontoparietal electrodes for nontraumatic MCS and UWS. NT=nontraumatic, T=traumatic.

When the role of brain injury aetiology was considered, nontraumatic MCS patients had a P3a that peaked in the frontal and temporal lobes with the presentation of emotional stimuli (Fig. 3A, C). Additionally, P3a was similarly implicated in nontraumatic UWS subjects (Fig. 3A). There was also a significant difference in P3a amplitudes between nontraumatic MCS and UWS participants.

In contrast, traumatic brain injury patients presented no peak P3a (Figure 3A). Moreover, the difference in P3a amplitude between the traumatic and nontraumatic groups was statistically significant (Fig. 3B).

### Time-varying networks

The above analysis of ERPs provided a background understanding of the timing of different stages during emotion stimulus processing. Given this background, we investigated the time course of varying network patterns across a 1000 ms period.

**Figure 4.**
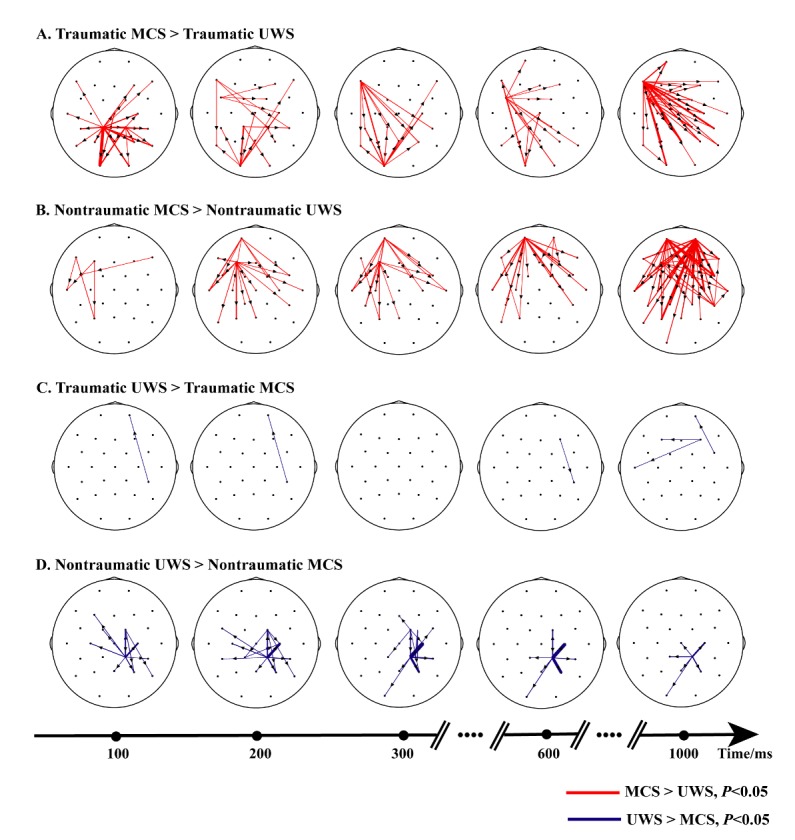
Significantly different network patterns in four conditions (rows) and for five time points (columns). Red lines illustrate increased connectivity in MCS compared to UWS, and blue lines illustrate decreased connectivity in MCS. The arrows indicate the direction of information flow. From 300 ms to 1000 ms, both traumatic MCS (A) and nontraumatic MCS (B) showed increased frontoparietal connectivity. (C) and (D) denote networks with stronger connectivity in UWS than MCS patients; the results from trauma patients are presented in the upper row (C), and those from nontraumatic UWS patients with occipital activation are in the bottom row (D).

In traumatic MCS patients, emotional sounds resulted in increased flow from the occipital region to the temporal and parietal regions between 100 and 200 ms; at 300 ms, occipital cortex activation and enhanced flow from the left frontal region to the parieto-occipital region were observed, lasting until 1000 ms (Fig. 4A). For nontraumatic MCS, no significantly increased information flow was detected at the early processing stage. From 200 to 1000 ms, differences were observed in the retrograde connections from the frontal region to the superior temporal and parietal regions. pecifically, nontraumatic MCS patients showed preserved top-down connectivity (Fig. 4B). In general, MCS patients exhibited increased top-down connectivity compared with UWS patients during the middle to late periods of emotional stimulus processing. In comparison, there was no significantly increased information flow in traumatic UWS patients (Fig. 4C), and information flow was altered only in the right occipital area in nontraumatic UWS patients (Fig. 4D).

**Figure 5.**
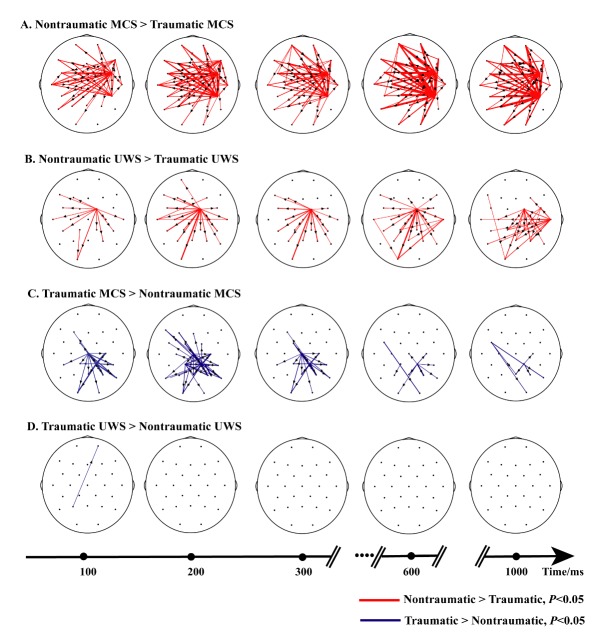
Time-varying network comparisons between traumatic and nontraumatic patients. Red lines illustrate increased connectivity in nontraumatic patients, and blue lines illustrate decreased connectivity. Increased connection from frontal regions to temporoparietal regions between 300 and 1000 ms was observed in nontraumatic MCS (A) and nontraumatic UWS (B). (C) Significant activation in the occipital area in traumatic MCS. (D) No significant increased network connectivity in traumatic UWS.

Next, we assessed the differences between patients with traumatic and nontraumatic conditions. Strengthened temporal activation in the early stage and increased expression of top-down influences from frontal regions to temporoparietal regions between 300 and 1000 ms were observed in nontraumatic MCS participants, especially after 600 ms, as illustrated by the thick lines (Figure 5A). In Figure 5B, increased activation was evident in the right primary motor cortex as well as between the frontoparietal lobes in nontraumatic UWS patients throughout emotional processing. In contrast, traumatic MCS patients exhibited increased activity only in the posterior areas, i.e., the occipital and parieto-occipital cortices (Fig. 5C), whereas no increase in information flow was detected in traumatic UWS patients (Fig. 5D).

### Correlation of network properties and CRS-R scores

**Figure 6.**
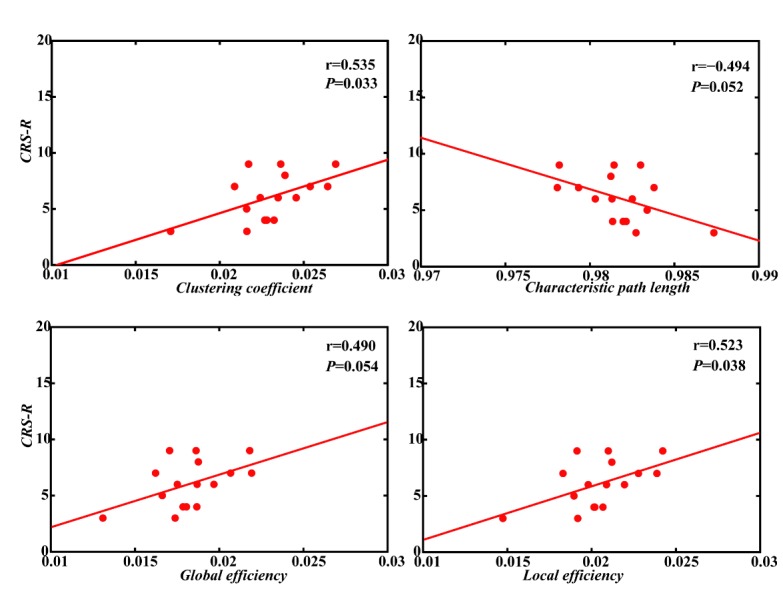
Traumatic patients showed a strong correlation between brain network properties and total CRS-R scores.

Here, four representative network properties, including the clustering coefficient (*C*), characteristic path length (*L*), global efficiency (*Ge*), and local efficiency (*Le*), were used to describe the strength of network connectivity. Among trauma patients, these network properties had significant linear correlations with CRS-R scores (*C*: *r* = 0.535, *P* = 0.033; *L*: *r* = -0.494, *P* = 0.052; *Ge*: *r* = 0.490, *P* = 0.054; *Le*: *r* = 0.523, *P* = 0.038; Fig. 6). However, there was no linear correlation between CRS-R scores and network properties in the nontraumatic group (C: r =0.081, *P* = 0.767; L: r = -0.121, *P* = 0.654; Ge: r = 0.120, *P* = 0.658; Le: r = 0.091, *P* = 0.738; Supplementary Fig. 2).

## DISCUSSION

In this study, we identified essential differences in both ERPs and dynamic network activation between MCS and UWS participants. Patients with UWS had impaired top-down processing compared with MCS patients, and the impaired frontoparietal connectivity in these individuals was usually accompanied by impairment of the higher-level cognitive abilities as assessed by middle to late ERP components. Specifically, such failures were evident in unconscious patients with traumatic brain injury.

### Distinct brain responses: ERP

The N1 component is thought to be an index of the pre-emotional perception of physical parameters of a stimulus (e.g., pitch, volume) [26]. Therefore, the evocation of N1 may suggest early automatic sensory identification among DOC patients. Another intriguing phenomenon was the prominent frontal P3a in nontraumatic MCS patients. It is generally hypothesized that P3a is a biomarker of exogenous attention and is triggered by “bottom-up” stimuli [27, 28]. In the present study, we found that involuntary, bottom-up attentional orientation may be preserved in DOC patients. However, some researchers have proposed that P3a may reflect the level of function of top-down attention switching, auditory attention, and an individual’s cognitive capability [29, 30]. Collectively, our results and the conclusions drawn by others consistently suggest that P3a reflects frontal attention.

On the basis of neuropharmacological and neurogenetic research, P3a has been described as a primarily dopaminergic ERP [31, 32]. Converging evidence from studies involving animals and humans has demonstrated that traumatic brain injury increases dopaminergic neurodegeneration [33]. Given this background, we hypothesized that the effect of traumatic brain injury on the dopaminergic system might account for the adverse interactions of the P3a waveform with cognition.

In addition to having connections with dopaminergic signalling, P3a is related to the uncertainty of an event [34]. A potential explanation is that increased attention is directed towards an infrequent stimulus to facilitate the modification of a behavioural response [35-37]. In healthy controls with intact feedback and regulatory systems, as in the present study, the experimental design, wherein deviant stimuli followed neutral prosody, allowed prediction and may thus have attenuated the amplitude of P3a or eliminated it entirely. Conversely, additional attentional resources were allocated to the higher-level cognitive tasks (as evidenced by evoked LPPs) [38, 39]. However, in MCS patients, as the infrequently presented emotional sounds can be regarded as a specific class of motivationally significant and attention-capturing stimuli, the additional attention increased the amplitude of the P3a component.

Additionally, various lines of evidence indicate that the frontoparietal junction affects the production of P3a and that frontal lobe engagement is therefore necessary for its appearance [40-42]. Hence, the present research explored the neural pathology of different patient groups in relation to P3a. Our work explored the brain network differences between those with and without a history of trauma exposure. The reduced frontoparietal activation in trauma-exposed patients may be related to degeneration of P3a. This topic is elaborated in further detail below in the discussion of network analysis.

LPP has been shown to increase significantly in response to emotional images and sounds. Thus, this component is established to be an index of emotion regulation processes [25, 43]. Available data suggest that LPP requires conscious recognition and sustained attention [43]. Specifically, this component is an index of high-level cognitive demands, such as memory encoding and storage [43, 44]. Hence, it is reasonable to speculate that high-level cognitive ability is greatly attenuated in DOC patients.

### Distinct brain network architecture

The present study demonstrated that the frontoparietal networks of patients with UWS were generally suppressed. Similar findings have been reported by Boly et al., who reported selective disruption of top-down processes from frontal to parietal regions in UWS patients using dynamic causal modelling [14]. Other single-modality studies consistently demonstrated that in patients with UWS, metabolic activity, along with functional and structural connectivity, is greatly reduced across widespread regions in the frontoparietal networks [11, 45-47]. These findings, in combination with our results showing an obvious dynamic reduction in frontoparietal connectivity during affective processing in patients with UWS, indicate that structural integrity may be linked to effective functional connectivity [48]. Further evidence of this link should be provided with multimodal studies that involve assessments of white matter structural integrity, metabolic function, and dynamic, temporally varying networks.

The present study explored brain network differences according to DOC aetiology. The disrupted functional connectivity of brain network architecture in trauma-exposed patients was significant here. On this basis, it is reasonable to suspect that the disrupted networks are associated with discrepant mechanisms of brain impairment. Diffuse axonal injury is the main mechanism underlying the effects of traumatic brain injury (TBI). Several studies have suggested that TBI may trigger persistent neurodegenerative processes [49]. Disruptions of functional connectivity in these individuals may be related to deficits in structural integrity [50], which also account for the decreased top-down processing. Another interesting finding is the obvious activation of the occipital lobes in trauma-exposed participants, supporting the compensatory role of the occipital areas in frontal- and parietal-lobe-driven cognitive processes. Occipital cortical activity is thought to indicate automatic or controlled cognitive processes, even in the absence of visual stimulation [51, 52]. This activity is believed to have a major influence on the processing of bottom-up input while being a suitable candidate for top-down processes [53, 54]. That is, top-down processing could act as a “gate”, controlling occipital cortical processing [55].

Importantly, no strong relationship between CRS-R scores and network properties existed in nontraumatic DOC patients. Considering this observation, we assert that the value of this behavioural assessment in patients without trauma needs to be re-evaluated, and additional criteria should be developed for the more accurate assessment of consciousness in these individuals. From another perspective, the network properties were not effective in identifying the remaining consciousness in nontraumatic DOC patients, whereas in the traumatic groups, these properties could reveal the levels of consciousness and further synergize with CRS-R evaluation.

There are some limitations to the present study. First, positive and negative affective stimuli were not analysed separately, a possible concern given the innate negativity bias [56]. In addition, diagnoses of MCS and UWS in this study cohort were based on the CRS-R scores, which may not accurately assess an individual’s state of consciousness. Diagnostic errors are worth considering, even if two clinicians performed these assessments daily for a week before participant enrolment to assess condition authenticity and stability. Moreover, in the present study, we did not localize brain injuries. Because of the confines of the bony ridges of the inner skull, focal cortical contusions resulting from inertial forces frequently cause damage localized in frontal and anterior temporal areas [57]. Additionally, structural brain images for all patients were obtained beforehand to exclude patients with significant brain tissue defects involving more than 30% of the total brain volume. It would be highly meaningful to divide the study groups according to lesion location; however, such stratification in our study would lead to inaccurate results because of the limited number of individuals in each group. In the future, studies with large sample sizes should be conducted to yield conclusive findings. Due to the inferior spatial resolution of the EEG method, developing a multidomain method combining EEG and MRI appears to be promising, since neuroimaging studies provide excellent spatial resolution to detect specific cortical areas associated with consciousness and emotional processing. Notably, we did not pay attention to the differences between DOC patients and healthy controls. Compared to healthy controls, DOC patients had conspicuous abnormalities in functional connectivity; nevertheless, the contrast between MCS and UWS was subtle and not observable. Specifically, the evoked LPP in healthy controls proved that such a simple emotional paradigm was associated with significant affective valence. Hence, in light of the failure to gaze in DOC, a novel sound stimulus or even a multi-modal stimulus is recommended, considering the perceptual enhancement that ?results from presenting stimuli in different modalities [58].

## Supplementary Materials

The Supplemenantry data can be found online at: www.aginganddisease.org/EN/10.14336/AD.2019.0606.
