# Supplementary material for: Impaired Frontoparietal Connectivity in Traumatic Individuals with Disorders of Consciousness: A Dynamic Brain Network Analysis
Source: Aging Dis. 2020 Mar 9;11(2):301–14. doi: 10.14336/AD.2019.0606 (PMC7069467; doi:10.14336/AD.2019.0606)
Supplement: Supplementary file 1 — The Supplemenantry data can be found online at: www.aginganddisease.org/EN/10.14336/AD.2019.0606. [file AD-11-2-301-s.pdf]

SUPPLEMENTARY DATA

Impaired Frontoparietal Connectivity in Traumatic Individuals with Disorders of Consciousness: A Dynamic Brain Network Analysis

Min Wu[1,#], Fali Li[2,#],Yuehao Wu[1], Tieying Zhang[1], Jian Gao[3], Peng Xu[2,*], Benyan Luo[1, *]

[1]Department of Neurology & Brain Medical Centre, First Affiliated Hospital, School of Medicine, Zhejiang University, Hangzhou, China.
[2]The Clinical Hospital of Chengdu Brain Science Institute, Key Lab for NeuroInformation, University of Electronic Science and Technology of China, Chengdu, China.
[3]Department of Rehabilitation, Hangzhou Hospital of Zhejiang Armed Police Corps, Hangzhou, China.

© 2019. Wu M et al. Published online at http://www.aginganddisease.org/EN/10.14336/AD.2019.0606

SUPPLEMENTARY DATA

METHODS

1. Time-varying multivariate adaptive autoregressive (TV-MVAAR) model

For each time series, the TV-MVAAR model was calculated with the following equation:

$$X(t) = \sum_{i=1}^{p} A(i,t)\,\mathrm{X}(t-i) + \mathrm{E}(t) \qquad (1)$$

where $X(t)$ is the data vector over the whole time window, $A(i,t)$ is the matrix of the TV-MVAAR model coefficients estimated by the Kalman filter algorithm, $E(t)$ is the multivariate independent white noise, and p is the model order, which can be automatically determined from the Akaike Information Criterion (AIC) with a range of 2 to 20:

$$AIC(\mathrm{P}) = \ln[\det(\Sigma) + 2M^2 p/N] \qquad (2)$$

where M is the number of the time series, p is the optimal model order, N is the time point and Σ is the covariance matrix. The observation and state equations were then solved by a recursive least-squares algorithm with a forgetting factor.

2. ADTF

Based on the time-varying coefficients estimated from (1), $H(f,t)$ can be further derived from the frequency domain expression of $A(i,t)$. The H_{ij} element of $H(f,t)$ represents the directed information flow from the j-th to the i-th element for each time point t:

$$A(f,t)X(f,t) = E(f,t) \qquad (3)$$

$$X(f,t) = A^{-1}(f,t)E(f,t) = H(f,t)E(f,t) \qquad (4)$$

where $A(f,t) = \sum_{k=0}^{p} A_k(t)\,\mathrm{e}^{-j2\pi f \square tk}$ is the frequency domain expression of model coefficients at time point t, and $X(f,t)$ and $E(f,t)$ are the transformations of $X(t)$ and $E(t)$ in the frequency domain.

The normalized ADTF describing the directed information flow from the j-th to the i-th element is defined as with the ADTF values between (0, 1):

$$r_{ij}^2(f,t) = \frac{\left|H_{ij}(f,t)\right|^2}{\sum_{m=1}^{n}\left|H_{im}(f,t)\right|^2} \qquad (5)$$

The integrated ADTF is defined as the average ADTF values over the frequency bands of interest:

$$\Theta_{ij}^2(t) = \frac{\sum_{k=f_1}^{f_2} r_{ij}^2(k,t)}{f_2 - f_1} \qquad (6)$$

Further details on the ADTF method can be found in previous studies [1]. Considering the frequency band of interest in emotion regulation, 0.1-30 Hz was selected as the range of the average ADTF values to serve as the directed information flow [2,3].

RESULTS

Figure S1. A contains the grand average ERPs at three midline electrode sites (Fz, Cz, and Pz) in the healthy control group. The N1 waveforms at these three electrodes for two different stimuli types were most prominent in frontotemporal areas. The typical LPP complex at Pz, ranging from 400 to 1000 ms, was evoked by affective prosody stimuli. The scalp distribution of the waveform peaks is displayed in Figure S1.B and demonstrates that the LPP peaks over parietal-occipital and central sites.

© 2019. Wu M et al. Published online at http://www.aginganddisease.org/EN/10.14336/AD.2019.0606

SUPPLEMENTARY DATA

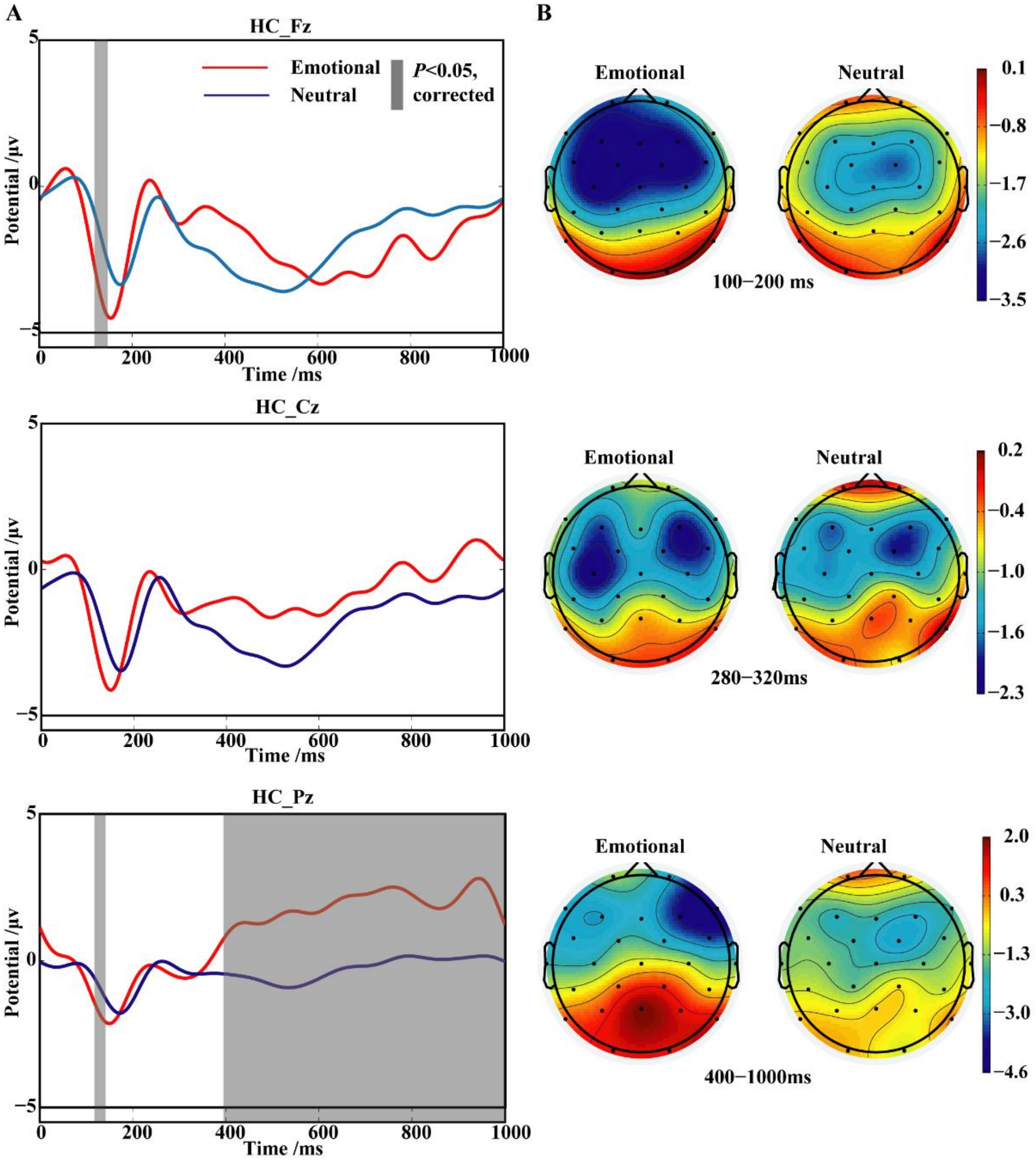

Supplementary Figure 1. **Grand average ERP components in healthy controls.** Grey bars indicate regions of significant difference between conditions (*P*<0.05, FDR corrected). (A) At electrode Fz, emotional sound evoked increased N1, and at Pz, an emotion-evoked LPP was observed. (B) The scalp topography during the three time periods of interest, corresponding to N1, P3a, and LPP, respectively.

© 2019. Wu M et al. Published online at http://www.aginganddisease.org/EN/10.14336/AD.2019.0606

SUPPLEMENTARY DATA

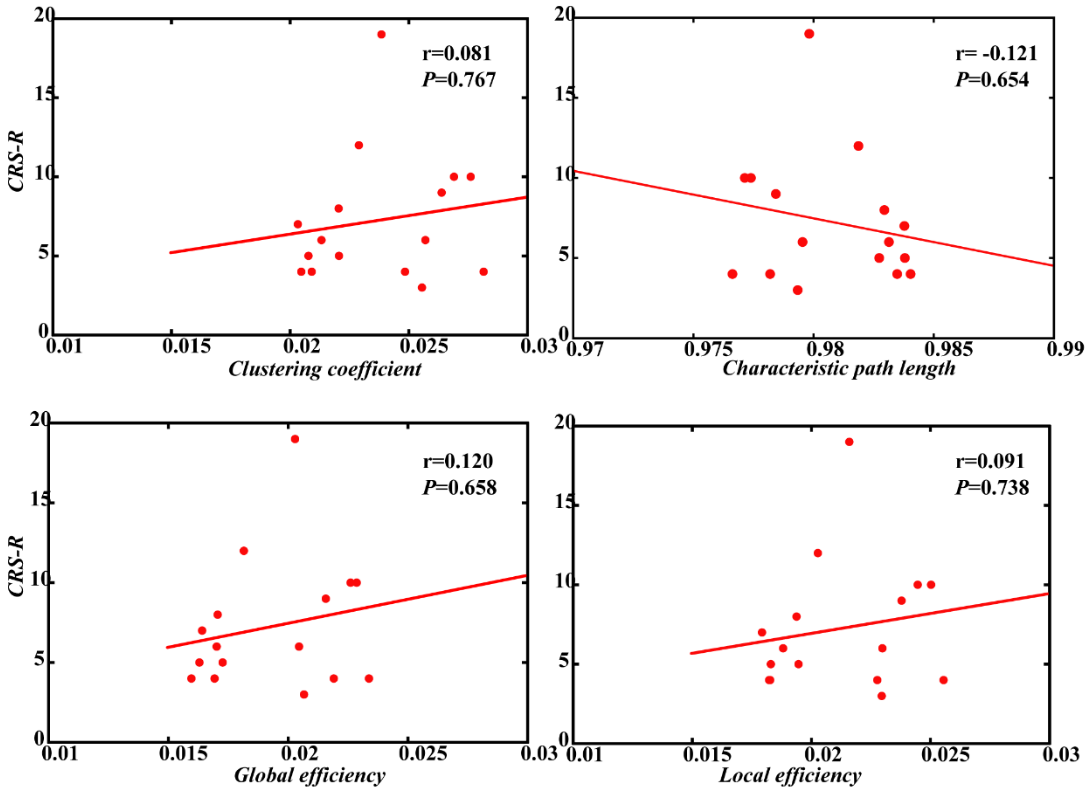

Supplementary Figure 2. No significant linear correlations existed between brain network properties and CRS-R total scores in patients with nontraumatic DOC.

References

[1] Wilke C, Ding L, He B (2008). Estimation of time-varying connectivity patterns through the use of an adaptive directed transfer function. IEEE Trans Biomed Eng, 55:2557-64.

[2] Brown DR, Cavanagh JF (2017). The sound and the fury: Late positive potential is sensitive to sound affect. Psychophysiology, 54:1812-25.

[3] Iredale JM, Rushby JA, McDonald S, Dimoska-Di MA, Swift J (2013). Emotion in voice matters: neural correlates of emotional prosody perception. Int J Psychophysiol, 89:483-90.

© 2019. Wu M et al. Published online at http://www.aginganddisease.org/EN/10.14336/AD.2019.0606
